# FCGR2C: An emerging immune gene for predicting sepsis outcome

**DOI:** 10.3389/fimmu.2022.1028785

**Published:** 2022-12-02

**Authors:** Si Liu, Yao Lu Zhang, Lu Yao Zhang, Guang Ju Zhao, Zhong Qiu Lu

**Affiliations:** ^1^ Emergency Department, The First Affiliated Hospital of Wenzhou Medical University, Wenzhou, Zhejiang, China; ^2^ Special Medical Department, Nanchong Central Hospital, Nanchong, Sichuan, China

**Keywords:** sepsis, immune genes, prognosis, FCGR2C, prognostic biomarker

## Abstract

**Background:**

Sepsis is a life-threatening disease associated with immunosuppression. Immunosuppression could ultimately increase sepsis mortality. This study aimed to identify the prognostic biomarkers related to immunity in sepsis.

**Methods:**

Public datasets of sepsis downloaded from the Gene Expression Omnibus (GEO) database were divided into the discovery cohort and the first validation cohort. We used R software to screen differentially expressed genes (DEGs) and analyzed DEGs’ functional enrichment in the discovery dataset. Immune-related genes (IRGs) were filtered from the GeneCards website. A Lasso regression model was used to screen candidate prognostic genes from the intersection of DEGs and IRGs. Then, the candidate prognostic genes with significant differences were identified as prognostic genes in the first validation cohort. We further validated the expression of the prognostic genes in the second validation cohort of 81 septic patients recruited from our hospital. In addition, we used four immune infiltration methods (MCP-counter, ssGSEA, ImmuCellAI, and CIBERSORT) to analyze immune cell composition in sepsis. We also explored the correlation between the prognostic biomarker and immune cells.

**Results:**

First, 140 genes were identified as prognostic-related immune genes from the intersection of DEGs and IRGs. We screened 18 candidate prognostic genes in the discovery cohort with the lasso regression model. Second, in the first validation cohort, we identified 4 genes (CFHR2, FCGR2C, GFI1, and TICAM1) as prognostic immune genes. Subsequently, we found that FCGR2C was the only gene differentially expressed between survivors and non-survivors in 81 septic patients. In the discovery and first validation cohorts, the AUC values of FCGR2C were 0.73 and 0.67, respectively. FCGR2C (AUC=0.84) had more value than SOFA (AUC=0.80) and APACHE II (AUC=0.69) in evaluating the prognosis of septic patients in our recruitment cohort. Moreover, FCGR2C may be closely related to many immune cells and functions, such as B cells, NK cells, neutrophils, cytolytic activity, and inflammatory promotion. Finally, enrichment analysis showed that FCGR2C was enriched in the phagosome signaling pathway.

**Conclusion:**

FCGR2C could be an immune biomarker associated with prognosis, which may be a new direction of immunotherapy to reduce sepsis mortality.

## Introduction

Sepsis is an acute and life-threatening syndrome with multiple organ dysfunction due to the host’s dysregulated response to infection (1). Although early and rational antibiotics, advanced supportive treatment, and high-quality care are essential in improving sepsis prognosis, sepsis mortality is still high (2, 3). Shockingly, more than 70% of septic patients die during the immunosuppressive phase of sepsis (4), which is caused by functional defects due to innate immune cells such as neutrophils, antigen-presenting cells(APCs) and adaptive immune cells such as T lymphocytes (5–7). These findings indicate that sepsis mortality is associated with immunosuppression and immune cell disturbance. Although immunosuppression of sepsis has been reported in many kinds of research, there are few studies on the value of immune molecules for predicting the prognosis of sepsis (8, 9). Therefore, there is still an urgent need to identify new immune markers to evaluate the prognosis of sepsis.

Recently, bioinformatics analyses have been widely used to identify immune infiltration markers of diseases (10–15). Immune cell infiltration is initially a pathological manifestation. Infiltrating cells are abnormal cells that should not occur in human tissues or the body under normal conditions (16). Microenvironment cell populations counter (MCP-counter) can use transcriptomic data to estimate the relative abundance of diverse immune and stromal populations in heterogeneous bulk samples (11). Single-sample gene set enrichment analysis (ssGSEA) can assess the score of immune cells and immune function (12). Immune Cell Abundance Identifier (ImmuCellAI) is based on the ssGSEA algorithm and focuses on the abundance of T-cell subtypes (13). MCP counter, ssGSEA, and ImmuCellAI were initially established for the immune infiltration analysis of tumor diseases (14, 15). However, some scholars still applied these three methods to study immune cells in non-tumor diseases, such as after carotid endarterectomy (17), endometriosis (18), and carotid artery atherosclerosis (19). Besides, many studies have used the CIBERSORT algorithm to evaluate the immune cell subtype distribution of tumor diseases and non-tumor diseases, such as osteoarthritis (20), acute myocardial infarction (21), skin diseases (22), and sepsis (8).

In this study, we divided datasets from the Gene Expression Omnibus (GEO) database into discovery and validation datasets. R software was used to screen differentially expressed genes (DEGs) and analyze DEGs’ functional enrichment in the discovery dataset. Then, the intersecting genes of the DEGs and IRGs were screened. The least absolute shrinkage and selection operator (LASSO) regression was used to identify vital immune candidate prognostic genes from the intersecting genes. Prognostic genes were identified from candidate prognostic genes in the first validation cohort. In addition, we validated the expression of the prognostic genes in the validation cohort. In the discovery dataset, we also applied four methods(MCP-counter, ssGSEA, ImmuCellAI, and CIBERSORT) to analyze immune cell composition infiltration in septic patients. In conclusion, this study aimed to provide novel immune prognostic biomarkers for sepsis.

## Materials and methods

### Data acquisition and processing

**Figure 1:**
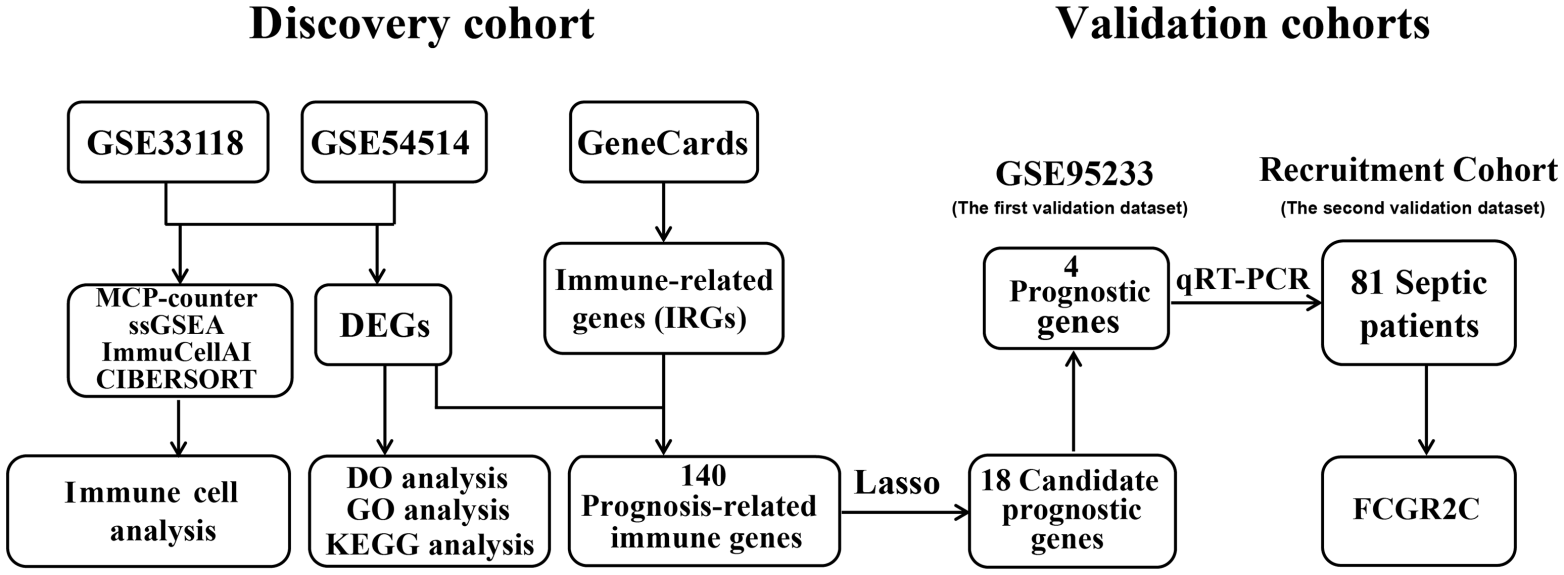
Flowchart of the study.

As shown in **Figure 1**, the sepsis RNA expression datasets GSE33118, GSE54514, and GSE95233 were downloaded from the GEO database with the “GEOquery” package in R software. The information on all public datasets is provided in **Table S1**. GSE33118 contained 20 whole blood samples, including 10 septic survivors and 10 non-survivors. GSE54514 included 35 whole blood samples, including 26 septic survivors and 9 non-survivors. GSE33118 and GSE54514 were merged into a cohort as the discovery dataset after batch effect correction (**Figure S1**). GSE95233 contained 51 whole blood samples, including 34 septic survivors and 17 non-survivors. The GSE95233 dataset was designated as the first validation dataset. All microarray datasets were background adjusted, and the data were normalized with the RMA algorithm. The batch effect of different datasets was removed by the “SVA” package (20).

### The information on recruitment cohort

In the present study, patients with documented or suspected infection plus an acute increase of ≥ 2 sequential organ failure assessment (SOFA) points were recorded as sepsis (1). Patients who met the consensus standards of Sepsis-3 and were hospitalized at the first affiliated hospital of Wenzhou Medical University from May 2021 to May 2022 were recruited. Patients under 18 or without signing the informed consent were excluded from this study. Patients were divided into septic survivors and non-survivors according to 90-day mortality. As the second validation cohort, we recruited 81 septic patients, including 53 survivors and 28 non-survivors. The Institutional Review Board of the First Affiliated Hospital of Wenzhou Medical University approved the study.

In addition, all patient clinical data were recorded, including demographics, the treatment during hospitalization, laboratory examination indicators within 24 h (including inflammatory and immune factors), Glasgow coma scale (GCS) score, SOFA score, and acute physiology and chronic health evaluation II (APACHE II) score.

### DEGs and IRGs screening

First, we divided the septic patients into septic survivors and non-survivors from the clinical information provided in the GEO datasets (**Table S1**). Then, we used the R package to differentially expressed genes (DEGs) of septic survivors and non-survivors patients, and DEGs were supposed to as prognostic-related genes. DEGs were identified with the “limma” R package (fold change(FC)> 1 or FC<1 and adj.P.value< 0.05) (23). IRGs were selected by searching the GeneCards website with the term “immune” (24). The GeneCards website will provide the relevance scores between these genes and immune. The higher the correlation score, the more it indicates that the gene is related to the immune. Prognostic immune genes were identified from the intersection of DEGs and IRGs.

### Prognostic biomarker screening and verification

In the discovery dataset, the LASSO regression model was used to identify significant candidate prognostic markers of sepsis through the “glmnet” package in R. All candidate prognostic genes were validated in the first validation dataset, and the genes with significant differences were identified as prognostic genes. Besides, we used the area under the curve (AUC) value to assess the diagnostic effectiveness through the “ROCR” R package in both the discovery and validation datasets.

### Functional enrichment analysis

We used the “DOSE”, “org.Hs.eg.db”and “clusterProfiler” R packages to perform Disease Ontology (DO), Gene Ontology (GO), and Kyoto Encyclopedia of Genes and Genomes (KEGG) enrichment analyses and visualize the enriched DO, GO, and KEGG pathways.

### Evaluation of immune cell in sepsis

MCP-counter is the first validated deconvolution method that calculates the abundance of 8 immune and 2 stromal cell populations using the transcriptome of cellularly heterogeneous tissues such as normal and malignant tissues (11). MCP-counter analysis was implemented with the “MCPcounter” R package. ssGSEA used the gene set related to the immune cell marker to assess the scores of 16 immune cells and 13 immune functions in tissues with the “gsva” R package (12). ImmuCellAI (http://bioinfo.life.hust.edu.cn/ImmuCellAI/), a website for analyzing 18 T cells and 6 other types of immune cells based on the ssGSEA algorithm, was also used. ImmuCellAI was applied to microarray and RNA-Seq expression profiles from various resources (e.g., tumor, adjacent or normal tissue, and peripheral blood) (13). CIBERSORT (https://cibersort.stanford.edu/index.php), a web analysis tool for evaluating the abundances of 22 immune cells in a mixed cell population, including peripheral blood samples, was used by inputting the gene expression data (25). Samples with P <0.05 were selected by the CIBERSORT algorithm.

### Quantitative Real-Time PCR (qRT-PCR)

First, samples were prepared by adding 1 ml of whole blood plus 200 μl of TRIzol Reagent (Invitrogen). Next, total RNA was extracted according to the manufacturer’s instructions. Then, 1 µg of total RNA was reverse transcribed using a Takara kit (RR047A). Candidate genes were assayed by qRT-PCR with a Thermo Fisher Scientific kit (A25742) on an Applied Biosystems QuantStudio™ 3 real-time PCR instrument (ABI). Relative gene expression levels were determined *via* the 2^−ΔΔCt^ formula and normalized to the expression of the internal control gene β-actin. The sequences of the gene-specific primers used in this study are shown in **Table S2**.

### Agarose electrophoresis and generation sequencing

The buffer was added to the qRT-PCR products to make a mixture, and then 10 ul of the mixture was added to the 2% agarose gel wells. Electrophoresis was performed at 120 v for 30 min, and the agarose gel was imaged with Amersham Imager 680(USA). Shanghai Biotech Biological Corporation(China) will do the generation sequencing of qRT-PCR products with an ABI sequencer (3730xlDNAAnalyzer, USA). A total of 4 files were obtained for the sequencing results of 1 qRT-PCR product, which were the sequence files (2 doc files) and the peak map files (2 ab1 files) for the forward and reverse strands. The first-generation sequencing results were displayed using SnapGene software(Version 4.3.6).

### Statistical analysis

All data were analyzed by R software 4.0.3. Continuous and classified variables were presented as mean ± standard deviation and number, respectively. Wilcoxon test and T-test was used to compare continuous variables, and the chi-square test was applied to compare categorical variables. Correlation analysis was performed using Pearson’s analysis. The data were statistically significant with P < 0.05 (*: P< 0.05, **: P< 0.01, ***: P < 0.001, ns:no significant).

## Results

### Identification of prognostic-related immune genes in sepsis

**Figure 2:**
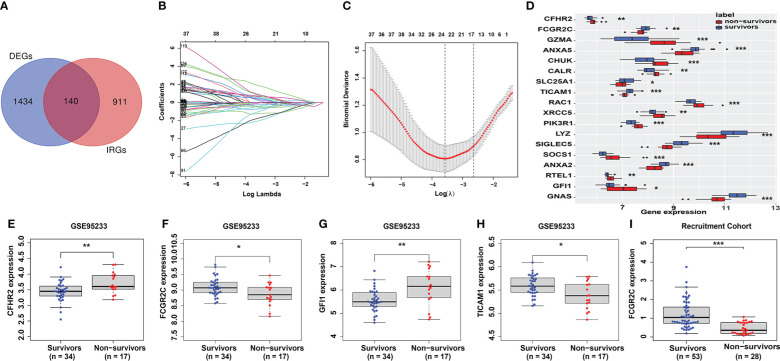
Identification and verification of prognostic genes. **(A)** The intersecting genes of DEGs and IRGs. **(B, C)** The Lasso coefficient values of 18 candidate prognostic genes were identified from the intersected genes in the discovery dataset. The vertical dashed lines are at the optimal log (λ) value. **(D)** The 18 candidate prognostic genes are shown in the box plot. **(E–H)** Identification of prognostic genes in the first validation cohort (GSE95233). Only 4 genes (CFHR2 **(E)**, FCGR2C **(F)**, GFI1 **(G)**, and TICAM1 **(H)**) had the same expression differences as those in the discovery cohort. **(I)** Verification of FCGR2C in the second validation cohort (our recruitment cohort). The expression difference of FCGR2C was the same as that in the discovery and first validation cohorts. (*:P< 0.05, **:P< 0.01, ***:P < 0.001).

In the discovery dataset, 157 genes were identified as DEGs (**Table S3**). Then, 1051 IRGs with a relevance score of more than 10 were included in this study (**Table S3**). Finally, 140 genes (**Table S3**) were identified as prognostic-related immune genes through the intersection of DEGs and IRGs (**Figure 2A**).

### Identification and verification of the prognostic genes

**Figure 3:**
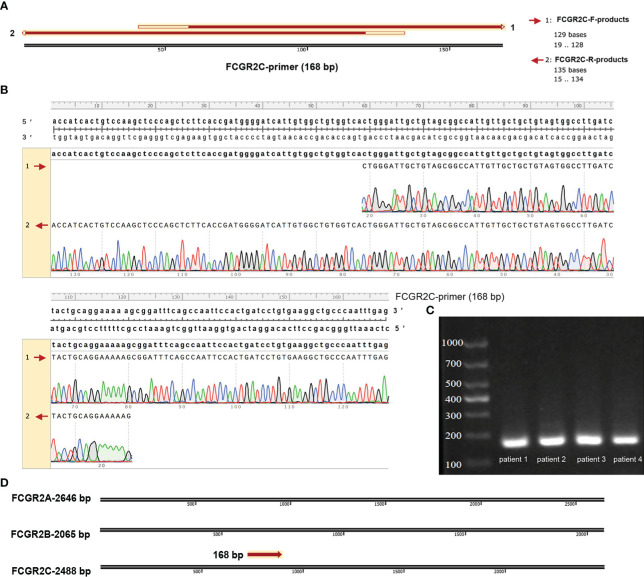
Validation of the qRT-PCR products of FCGR2C. **(A)** Matching the forward and reverse strand product plots with the primer sequences specific to FCGR2C. **(B)** Details of the complementary base pairing of the forward and reverse strand products with the FCGR2C primer sequence. **(C)** Agarose electrophoresis plot of qRT-PCR amplification products of FCGR2C. **(D)** The 168 base sequence is specific to FCGR2C. First-generation sequencing is a double-end sequencing of the product based on the forward and reverse strand sequences of the primer. The forward strand sequencing results of the products are indicated by 1. The reverse strand sequencing results of the products are indicated by 2. The primers of FCGR2C were designed with a length of 168 bp. The agarose electropherogram showed only one band consistent with the primer sequence’s size, indicating the primer’s specificity. In this study, we performed generation sequencing and agarose electrophoresis of qRT-PCR products from septic patients. Some of the patients’ results are shown in the pictures. The sequencing results of the forward and reverse sequences of qRT-PCR products and the sequence files after double-end splicing are shown in **Table S6**.

The Lasso regression model (**Figures 2B, C**) screened 18 candidate genes (**Figure 2D**; **Table S3**) from 140 prognostic-related immune genes. However, in the first validation dataset, only 4 genes (CFHR2, FCGR2C, GFI1, and TICAM1) with differential expression were identified as prognostic genes (**Figures 2E–H**), and the other genes were not differentially expressed(**Figures S2A–N**). In the discovery and first validation cohorts, the AUC values for assessing the prognosis of sepsis by combining the 4 genes were 0.93 (**Figure S3A**) and 0.83(**Figure S3B**), respectively. Finally, in our recruitment cohort, FCGR2C was the only gene differentially expressed between the survivors and non-survivors of 81 septic patients (**Figure 2I** and **Figures S5A–C**). Unfortunately, FCGR2C was not differentially expressed in the discovery and validation cohorts in a different age, country, and sex groups (**Figures S4A–G**). In addition, agarose electrophoresis demonstrated the FCGR2C primers’ specificity (**Figure 3C**). Moreover, the first-generation sequencing results showed that the base sequences of the forward and reverse strands of the qRT-PCR amplification products matched precisely with the FCGR2C primer design of 168 bases(**Figures 3A, B**). Although FCGR2C is highly homologous with FCGR2A and FCGR2B, the 168 bases sequence matches only the FCGR2C gene sequence, not FCGR2A and FCGR2B(**Figure 3D**). Collectively, these findings suggest that FCGR2C is differentially expressed in sepsis.

### Correlation analysis of FCGR2C and clinical indicators

**Figure 4:**
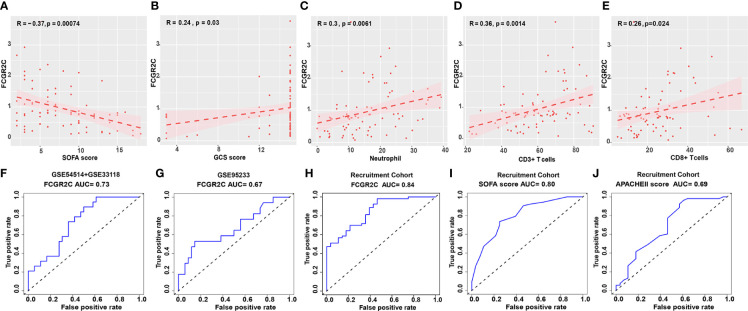
Clinical role of FCGR2C. Correlation analysis of FCGR2C with the SOFA score **(A)**, the GCS score **(B)**, neutrophils **(C)**, CD3+ T cells **(D)**, and CD8+ T cells **(E)**. **(C-E)**: The x-axis represents the absolute value of neutrophils, the percentage of CD3+ T cells, and CD8+ T cells, respectively. **(F)** The prognostic evaluation ability of FCGR2C in the discovery dataset (GSE54514+GSE33118). **(G)** The prognostic evaluation ability of FCGR2C in the first validation dataset (GSE95233). The prognostic evaluation ability of FCGR2C **(H)**, the SOFA score **(I)**, and the APACHE II score **(J)** in the second validation cohort (our recruitment cohort).

The clinical information of the recruited septic patients in this study is shown in **Table S4**. According to **Table S4**, most septic non-survivors used invasive ventilators and hemodialysis during the treatment process. In the recruitment cohort, we found that the levels of the SOFA score, APACHE II score, and IL-2, IL-6, IL-10, and TNF-α in septic non-survivors were significantly higher than those in survivors (**Table S4**). In comparison, the values of neutrophils, the GCS score, CD3+ T cells, CD8+ T cells, the Absolute value of T cells, the Absolute value of CD8+ T cells, and C3 were significantly lower (**Table S4**). To further study the role of FCGR2C in the clinic, we performed a correlation analysis between FCGR2C and clinical indicators. The results showed that FCGR2C was negatively correlated with the SOFA score (r=-0.37, P=0.00074, **Figure 4A**), but was positively correlated with the GCS score (r=0.24, P=0.03, **Figure 4B**), neutrophils (r=0.3, P=0.0061, **Figure 4C**), CD3+ T cells (r=0.36, P=0.0014, **Figure 4D**) and CD8+ T cells (r=0.26, P=0.024, **Figure 4E**). Thus, these findings indicated that FCGR2C might be a potential indicator that could help assess the condition of septic patients.

### Prognostic model evaluation of biomarkers in sepsis

In the discovery dataset, the AUC value of FCGR2C was 0.73 (**Figure 4F**). The AUC values of FCGR2C were 0.67 (**Figure 4G**) in the first validation dataset (GSE95233) and 0.84 (**Figure 4H**) in the second validation cohort (Recruitment cohort). In addition, we found that the predictive assessment ability of FCGR2C was superior to that of the SOFA score (AUC=0.80) and APACHE II score (AUC=0.69) (**Figures 4I, J**). In summary, FCGR2C had considerable prognostic significance for discriminating between septic survivors and non-survivors in the discovery and validation datasets.

### Differences in immune cells between septic survivors and non-survivors

**Figure 5:**
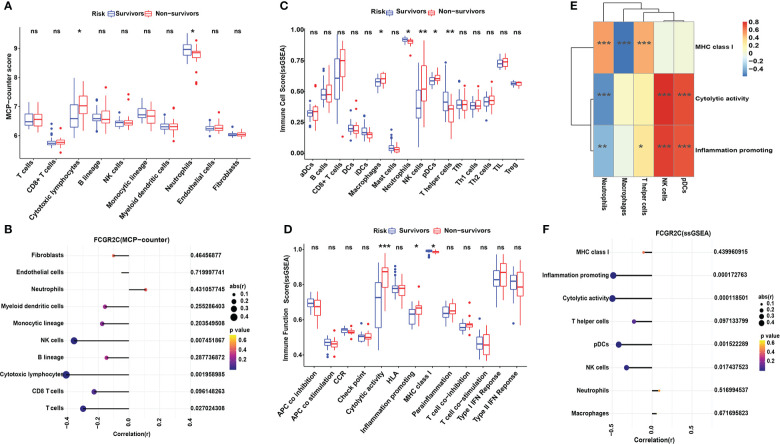
Immune cell analysis with MCP-counter and ssGSEA in the discovery cohort. **(A)** The MCP-counter algorithm was used to analyze the immune cell relative abundance in septic survivors and non-survivors. The MCP-counter analyzes 8 immune cell populations(T cells, CD8 T cells, cytotoxic lymphocytes, B lineage cells, NK cells, monocytic lineage cells, myeloid dendritic cells, and neutrophils) and 2 stromal cell populations (endothelial cells and fibroblasts). **(B)** Correlation analysis of FCGR2C with immune cells and stromal cells. ssGSEA was used to analyze the 16 immune cells **(C)** and 13 immune functions **(D)** between septic survivors and non-survivors. **(E)** Heatmap of the correlation between immune functions and immune cells by ssGSEA. Color changes show the correlation intensity; red indicates a positive correlation, and blue indicates a negative correlation. **(F)** Correlation analysis of FCGR2C with immune functions and immune cells. Dots indicate the power of the correlation, and different colors indicate the P-value. (*P< 0.05, **P< 0.01, ***P < 0.001, ns:no significant).

**Figure 6:**
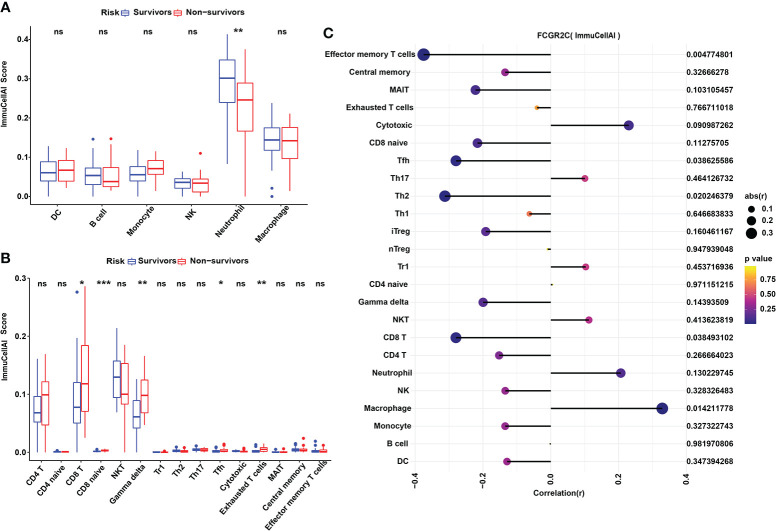
Immune cell analysis with ImmuCellAI in the discovery cohort. **(A)** The ImmuCellAI algorithm was used to analyze the immune cells other than T cells between septic survivors and non-survivors. **(B)** The ImmuCellAI algorithm was used to compare T-cell subsets between septic survivors and non-survivors. **(C)** Correlation analysis of FCGR2C with immune cells. Dots indicate the intensity of the correlation, and different colors indicate the P-value. (*P< 0.05, **P< 0.01, ***P < 0.001, ns:no significant).

MCP-counter was used to explore the abundance and immune score of immune and non-immune cells between the survivors and non-survivors groups. The results showed neutrophils had the highest immune score, with significant differences between septic survivors and non-survivors (**Figure 5A**). Similarly, the same results were obtained in ssGSEA (**Figure 5C**) and ImmuCellAI analysis (**Figure 6A**). From **Figure 5A**, cytotoxic lymphocytes notably differed between the septic survivors and non-survivors. However, there were no differences in T cells, CD8+ T cells, B lineage cells, monocytic lineage cells, myeloid dendritic cells, endothelial cells, or fibroblasts. Furthermore, we analyzed the correlations of FCGR2C with immune and stromal cells (**Figure 5B**). The results indicate that FCGR2C was negatively correlated with cytotoxic lymphocytes (r=-0.409, P=0.002), NK cells (r=-0.357, P=0.007) and T cells (r=-0.298, P=0.027).

ssGSEA was also used to calculate the enrichment scores of different immune cell subgroups and explore the immune functions between survivors and non-survivors. **Figure 5C** shows that macrophages, NK cells, pDCs, and T helper cells differed between the septic survivors and non-survivors. Moreover, immune functions, such as cytolytic activity, inflammation-promoting, and MHC class I, were different in septic non-survivors compared with survivors (**Figure 5D**). The results of the correlation analysis of immune cells and immune functions (**Figure 5E**) showed a significant negative correlation between macrophages and MHC class I (r=-0.56, P<0.001). Although septic non-survivors had an increased proportion of NK cells and pDC compared to survivors, both immune cell types were significantly and positively associated with cytolytic activity (r=0.829, P<0.001; r=0.756, P<0.001) and inflammatory promotion (r=0.775, P<0.001; r=0.725, P<0.001). Moreover, we analyzed the correlations of FCGR2C with immune cells and functions (**Figure 5F**). The results indicate that FCGR2C was negatively correlated with cytolytic activity (r=-0.496, P*<*0.001), inflammation promotion (r=-0.485, P*<*0.001), and NK cells in sepsis (r=-0.319, P=0.017).

In addition, we used ImmuCellAI to evaluate the abundance of T-cell subsets in sepsis. The relative abundances of immune cells are shown in **Figure S6A**. The results showed that the immune scores of Gamma delta (Tgd), Tfh, and Exhausted (Tex) T cells were higher in septic non-survivors than in survivors (**Figure 6B**). We also found that Tfh cells were positively correlated with Tex cells, and neutrophils were negatively correlated with T-cell subsets through correlation heatmap analysis (**Figure S6B**). Besides, we found that FCGR2C was negatively correlated with effector memory (Tem) T cells (r=-0.375, P=0.005), Tfh cells(r=-0.280, P=0.039), and Th2 cells(r=-0.312, P=0.02) but positively correlated with macrophage (r=0.29, P=0.014) (**Figure 6C**).

**Figure 7:**
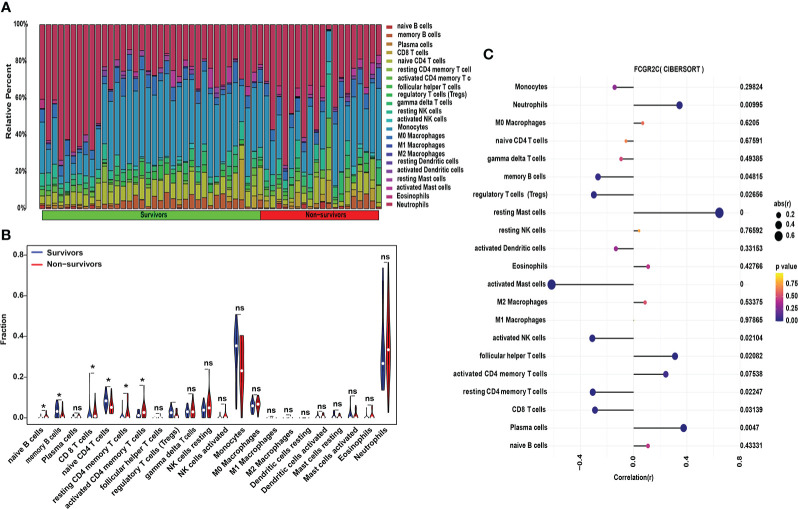
Immune cell analysis with CIBERSORT in the discovery cohort. **(A)** The relative percentages of 22 subpopulations of immune cells in septic patients. **(B)** The difference in immune cells between septic survivors and non-survivors. **(C)** Correlation analysis of FCGR2C with 22 immune cells. Dots indicate the intensity of the correlation, and different colors indicate the P-value. (*P< 0.05, ns: no significant.)

Also, we determined the profile of 22 immune cell subtype distribution patterns by the CIBERSORT algorithm. Although neutrophils and monocytes accounted for the most significant proportion of the 22 immune cells (**Figure 7A**), there was no significant difference between septic non-survivors and survivors (**Figure 7B**). From **Figure 7B**, we found that the proportions of naive B cells, memory B cells, naive T CD4 cells, resting CD4 memory T cells, and activated CD4 memory T cells differed between septic survivors and non-survivors. Subsequently, we found that M2 macrophages were positively correlated with follicular helper T cells by correlation heatmap analysis (**Figure S7A**). Interestingly, neutrophils showed a significant negative correlation with monocytes but also a significant positive correlation with plasma cells (**Figure S7A**). The PCA cluster analysis showed no clear distinction could be made between septic survivors and non-survivors based on immune cells (**Figure S7B**). Additionally, we found that FCGR2C was negatively correlated with memory B cells and activated mast cells but positively correlated with resting mast cells and plasma cells (**Figure 7C**). Collectively, these findings suggest that FCGR2C may be related to multiple immune cells.

### Functional enrichment analysis

**Figure 8:**
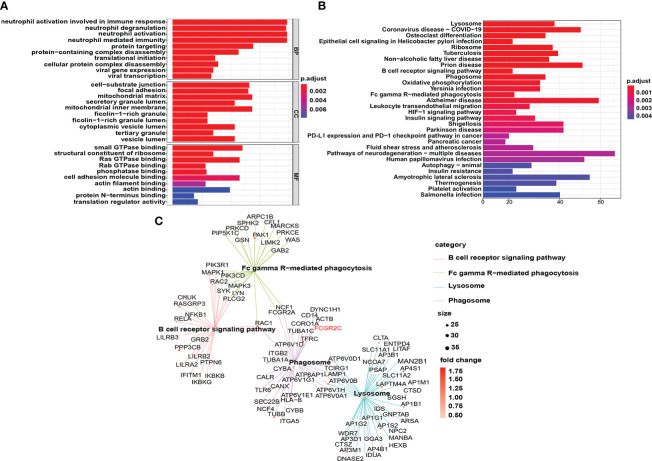
Functional enrichment analysis of DEGs in the discovery cohort. **(A)** The top 10 results of the cellular component (CC), molecular function (MF), and biological process (BP) categories in GO enrichment analysis are shown. **(B)** The top 30 KEGG enrichment results with the most significant P-values are displayed. **(C)** Genes enriched in the B-cell receptor signaling pathway, Fc gamma R-mediated phagocytosis, lysosome, and phagosome in the KEGG results are shown.

In the discovery dataset, we used R software to perform DO, GO, and KEGG analyses of the DEGs. The results of DO, GO, and KEGG analyses are shown in **Table S5**. The DO analysis results showed that the differential prognostic genes of sepsis were mainly enriched in disease by an infectious agent (**Figure S8**), which confirms that sepsis is triggered by infection. GO analysis indicated that the DEGs were primarily enriched in biological process (BP) pathways, such as neutrophil activation pathways involved in the immune response, neutrophil activation, and neutrophil-mediated immunity. Cellular component (CC) pathways mainly included the mitochondrial matrix, inner membrane, and cell-substrate junction. The molecular function (MF) pathways mainly had small GTPase binding, Ras GTPase binding, and cell adhesion molecule binding (**Figure 8A**).

The lysosome pathway had the most significant P value of the enriched KEGG pathways (**Figure 8B**). In addition, many phagocyte function pathways were enriched, such as the B-cell receptor signaling pathway, phagosome, and Fc gamma R-mediated phagocytosis. More importantly, FCGR2C is involved in the phagosome signaling pathway (**Figure 8C**), which confirms the close correlation between FCGR2C and immune cell function from another perspective.

## Discussion

Sepsis, a leading cause of death in the ICU, has a mortality rate of approximately 30% (26). Notably, sepsis mortality remains high and may be caused by immunosuppression (27). Thus, there is a need to find new methods to predict sepsis prognosis and immunity. Recently, it has been reported that biomarkers can predict sepsis prognosis (9, 28). Xu C et al. found that MMP9 and C3AR1 are associated with sepsis prognosis. Besides, CEBPB may be a critical immune gene in sepsis (8). However, the GEO datasets used in their research contained two kinds of RNA expression data of neutrophils and whole blood samples, which does not guarantee the authenticity of the results. Moreover, the study did not clarify the correlation between biomarkers and immune cells. Consequently, there is still an urgent need to find new practical immune markers to predict sepsis prognosis.

In the study, FCGR2C was identified as a prognostic immune biomarker. The Fc gamma receptor 2C gene (FCGR2C, FcγRIIC, CD32C) is a low-affinity Fc gamma receptor(FcγR). FcγRs are the cellular receptors in the Fc region of immunoglobulin G (IgG). FcγR is expressed on various types of cells, including macrophages, dendritic cells, NK cells, B lymphocytes, neutrophils, and platelets (29). At present, six kinds of FcγRs have been confirmed in humans, namely, one high-affinity receptor (FcγRI) and five low-to-medium-affinity receptors (FcγRIIA, -B and -C, and FcγRIIIA and -B). After binding to complex IgG, FcγRs can trigger various cellular immune response functions, thus connecting the adaptive and innate immune systems, ultimately destroying and eliminating conditioning targets (30). In 1998, Metes D et al. first identified four different products of FCGR2C in NK cells (31). FCGR3A is the receptor expressed in NK cells of all individuals and responsible for antibody-dependent cell-mediated cytotoxicity. In some individuals with FCGR2C-ORF haplotypes, FCGR2C may contribute something to this effect (31). In 2013, Li X et al. first identified allele-dependent expression of activated FCGR2C on B cells. Furthermore, they found that FCGR2C on B cells promotes humoral immunity in humans and mice (32). Consistent with the results of a previous study, we found that FCGR2C was significantly correlated with cytolytic activity and NK cells by ssGSEA. In summary, FCGR2C may be a crucial functional molecule in immune cells.

FCGR2C is a pseudogene, and it is only expressed on the surface of cells (mainly NK cells) of about 20% or so of individuals of European origin (white). It is not expressed in, for example, individuals from Thailand, black South Africans, among many other populations (33, 34). It is because they possess a splice variant that results in a lack of surface expression as a functional protein. Nevertheless, a growing number of researchers are now focusing on FCGR2C. Liu F et al. found that FCGR2C was the most significant DEG between normal and Sickle cell disease (SCD) in African Americans (35). Xu W et al. found that FCGR2C was one of the common genes in Nonalcoholic fatty liver disease (NAFLD) and periodontitis (36).

In this study, FCGR2C was differentially expressed between septic survivors and non-survivors in both discovery and validation cohorts. We speculate that this situation may be due to the increased susceptibility to immune dysfunction in septic non-survivor patients. Moreover, FCGR2C is related to genetic variation in immune and alloimmune diseases (30). Consistent with our study, Wang Y et al. found that FCGR2C was a significantly differential gene in extranodal NK/T-cell lymphoma (ENKTL) patients from West China Hospital in Sichuan, China (37). Nevertheless, Breunis WB et al. first reported that in 91% of the white control population, a single nucleotide polymorphism(SNP) mutation of exon 3 (rs759550223) resulted in the lack of functional expression of the FCGR2C in their immune cells (30, 38). Most FCGR2C alleles are not expressed due to novel splice site mutation of near exon 7 (29, 30, 39). Nagelkerke SQ et al. found that the classic FCGR2C-ORF haplotype was virtually absent in Chinese Children from Canada of Han-Chinese descent, all of which were grandparent-proven Han-Chinese (39). Hence, it needs to explore further the expression, SNP, and splice site mutation of FCGR2C in multi-center and multinational collaborative research populations of different ages. Because FCGR2A, FCGR2B, and FCGR2C have very high sequence homology, RNA expression data from public databases are therefore very vulnerable to artifacts in the sequencing and wrong allocation to either FCGR2A, FCGR2B, or FCGR2C. Additionally, these splice site mutations in FCGR2C and nonsense-mediated decay resulting from the stop codon in exon 3 in the majority of FCGR2C alleles, and the relative abundance of FCGR2A transcripts in circulating leukocytes may be much greater than FCGR2C transcripts. Although we used specific primers for qRT-PCR, agarose electrophoresis, and first-generation sequencing of qRT-PCR products, due to the limitations of existing samples and experimental techniques, we cannot completely rule out the possibility of non-specific amplification of FCGR2A in our study. Consequently, we will use advanced technology and multi-center clinical research to verify our results further in our next investigation.

Because of the very high homology between FCGR2A, FCGR2B, and FCGR2C, it is a difficult gene cluster to study. We found that the expression of FCGR2B had no difference in both discovery (**Table S3**) and validation cohorts (**Figures S2P**, **S5E**). Although FCGR2A was differentially expressed in the discovery cohort (**Table S3**) and our recruitment cohort(**Figure S5D**), there was no differential expression in the first validation cohort (**Figure S2O**). Bougle A et al. found that the homozygosity of FCGR2A-p.166Arg was independently associated with decreased hospital mortality in invasive pneumococcal diseases (IPDs) (40). The variant FCGR2A-p.166Arg may be a marker of genetic susceptibility to sepsis (41). Also, Solé-Violán J found that patients with bacteremic PCAP (B-PCAP) were associated with sepsis severity and homozygosity for FCGR2A-p.166His predisposes B-PCAP, which suggests that the variant FCGR2A-p.166His may be related to sepsis severity (42). Therefore, FCGR2A may be closely associated with sepsis severity, but it still needs to be confirmed by a multi-center and extensive sample study.

Interestingly, we first found that FCGR2C may be an emerging immune gene for predicting sepsis outcomes. Similarly, Ribeiro I P et al. found that FCGR2C can predict the survival of head and neck squamous cell carcinoma (HNSCC) patients from The Cancer Genome Atlas (TCGA) (43). Since FCGR2C may not be expressed due to splicing mutation, the predictive evaluation ability of FCGR2C in immune-related diseases should be explored in depth. In addition, we found that FCGR2C was not differentially expressed in different age, country, and gender groups in the discovery and validation cohorts. There are no references for studies between FCGR2C and age and gender. Therefore, studies on the expression levels of FCGR2C in different races, genders, and ages need to be confirmed by multi-center and multinational collaborative clinical studies.

Our study observed cytotoxic lymphocytes increased in septic non-survivors compared with the septic survivors. Consistent with our research, Napoli A M et al. found that the expression level of cytotoxic lymphocytes was increased in severe septic patients (44). Another study showed that CTLA-4, a molecule in immune cytotoxic lymphocytes, is up-regulated in sepsis, which can promote the occurrence of immunosuppression and eventually increase mortality (45). We found that FCGR2C was negatively correlated with cytotoxic lymphocytes by MCP-counter analysis. Therefore, we can infer that the low level of cytotoxic lymphocytes in septic survivors may be related to the high level of FCGR2C expression. FCGR2C may be an emerging factor that relates to cytotoxic lymphocyte function.

Immunosuppression in septic patients is caused by the depletion and loss of immune cells, including B cells, CD4+ T cells, and other important immune cells (46–48). The possible cause of increased mortality in late septic patients is immunosuppression. In addition, previous studies have demonstrated that decreasing the number of memory B cells can increase mortality in sepsis (47). Similarly, we found that the levels of memory B cells were reduced in septic non-survivors compared with septic survivors by CIBERSORT analysis. Additionally, we found that resting memory CD4 T cells and activated memory CD4 T cells were higher in septic non-survivors than in septic survivors. Nevertheless, FCGR2C was negatively correlated with resting memory CD4 T cells. Therefore, we speculate that FCGR2C may be related to transforming the resting and activated states of memory CD4 T cells. Furthermore, we found that FCGR2C was negatively correlated with T-cell subtypes, such as Tem, Tfh, and Th2 cells, by ImmuCellAI analysis. Also, FCGR2C was associated with robust dendritic cells (DC) and T-cells (37). Therefore, FCGR2C may be related to the functional maintenance of immune cells. The functional role of FCGR2C is a topic worth investigating.

In addition, we found that most DEGs were enriched in immune pathways regulated by neutrophils from GO and KEGG analyses. The results of our study are consistent with those of Xu C’s team (8). MCP-counter, ssGSEA, and ImmuCellAI analysis also showed that the high immune scores in septic patients were due to neutrophils. In addition, in the discovery and validation cohorts, neutrophils were significantly lower in septic non-survivors than in septic survivors. Moreover, FCGR2C was positively correlated with neutrophils in the discovery and our recruitment cohorts. KEGG analysis revealed that FCGR2C is a critical gene in the phagocytic signaling pathway. The phagocytic function is necessary for neutrophils to clear pathogens. Therefore, exploring the expression of FCGR2C in neutrophils and performing functional studies were worthwhile.

The SOFA score (49) and APACHE II score (50) are used to diagnose sepsis and evaluate its prognosis. Strikingly, our findings suggest that the predictive power of FCGR2C is higher than that of the SOFA score and APACHE II score based on the AUC values. Additionally, there was a significant negative correlation between FCGR2C and the SOFA score. This study showed that FCGR2C might be an immune marker with crucial clinical significance.

To the best of our knowledge, our study is the first to combine the GEO database and a new recruitment cohort to identify and validate immune prognostic markers between septic survivors and non-survivors. Moreover, we found that FCGR2C may be a prognostic immune indicator in sepsis. Nevertheless, many limitations remain in our research. First, although discovery and validation cohorts were used in this study, the number of septic patients was small, and the subjects’ race was limited. Second, we studied all the genes screened by LASSO regression in the first validation cohort. We then selected the genes with different expressions in the two cohorts as the targets for the following analysis. This research method may include genes excluded by other machine learning methods(such as Support Vector Machines) when screening features. Third, this study initially aimed to identify the prognostic biomarkers related to immunity in sepsis. The functional role of FCGR2C in sepsis requires more rigorous experiments to explore. Besides, there was a difference in CD8+ T cells between the recruitment cohort and the discovery cohort of septic patients. We found that CD8+ T cells were decreased in septic non-survivors compared with septic survivors, consistent with the findings of many reported studies (51, 52). Last, the 4 immune infiltration analysis methods were initially developed to analyze tumor tissues’ immune cells, so there may be differences in using these four analytical methods to assess immune cells in non-tumor diseases.

## Conclusion

In the study, FCGR2C may be more powerful than the SOFA score and APACHE II score in evaluating the prognosis of sepsis. FCGR2C could closely relate to a variety of immune cells and immune functions. In conclusion, FCGR2C may be a novel immune marker related to sepsis prognosis.

## Data availability statement

Publicly available datasets were analyzed in this study. This data can be found here: https://www.ncbi.nlm.nih.gov/geo/ - GSE33118, GSE54514 and GSE95233.

## Ethics statement

The studies involving human participants were reviewed and approved by ethics committee in clinical research of the First Affiliated Hospital of Wenzhou Medical University (2022129). The patients/participants provided their written informed consent to participate in this study. Written informed consent was obtained from the individual(s) for the publication of any potentially identifiable images or data included in this article.

## Author contributions

SL and YZ contributed equally to this work. LZ contributed to the subject recruitment and clinical information collection. ZL and GZ guided the conception of the article. All authors read and approved the final manuscript. All authors contributed to the article and approved the submitted version.

## Funding

This work was supported by Zhejiang Research and Development Projects (2021C03072), the National Key R&D Program of China (2018YFC2000305), the National Natural Science Foundation of China (81871583), the Natural Science Foundation of Zhejiang Province (LQ22H150003), and the Province Nanchong City School Science and Technology Strategic Cooperation Project (18SXHZ0454).

## Conflict of interest

The authors declare that the research was conducted in the absence of any commercial or financial relationships that could be construed as a potential conflict of interest.

## Publisher’s note

All claims in this article are solely those of the authors and do not necessarily represent those of their affiliated organizations or the publisher, the editors, or the reviewers. Any product that may be evaluated in this article, or claim made by its manufacturer, is not guaranteed or endorsed by the publisher.
