# Supplementary material for: FCGR2C: An emerging immune gene for predicting sepsis outcome
Source: Front Immunol. 2022 Dec 2;13:1028785. doi: 10.3389/fimmu.2022.1028785 (PMC9757160; doi:10.3389/fimmu.2022.1028785)
Supplement: Supplementary file 1 [file DataSheet_1.pdf]

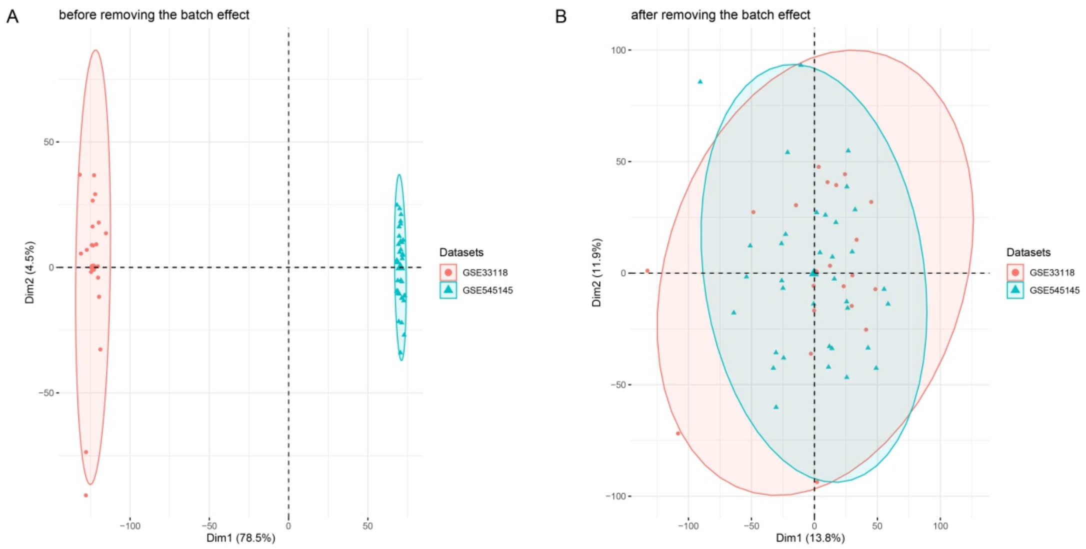

Figure S1 Removal of the batch effect of GSE54514 and GSE33118 using the “SVA” package: (A) before removing the batch effect; (B) after removing the batch effect.

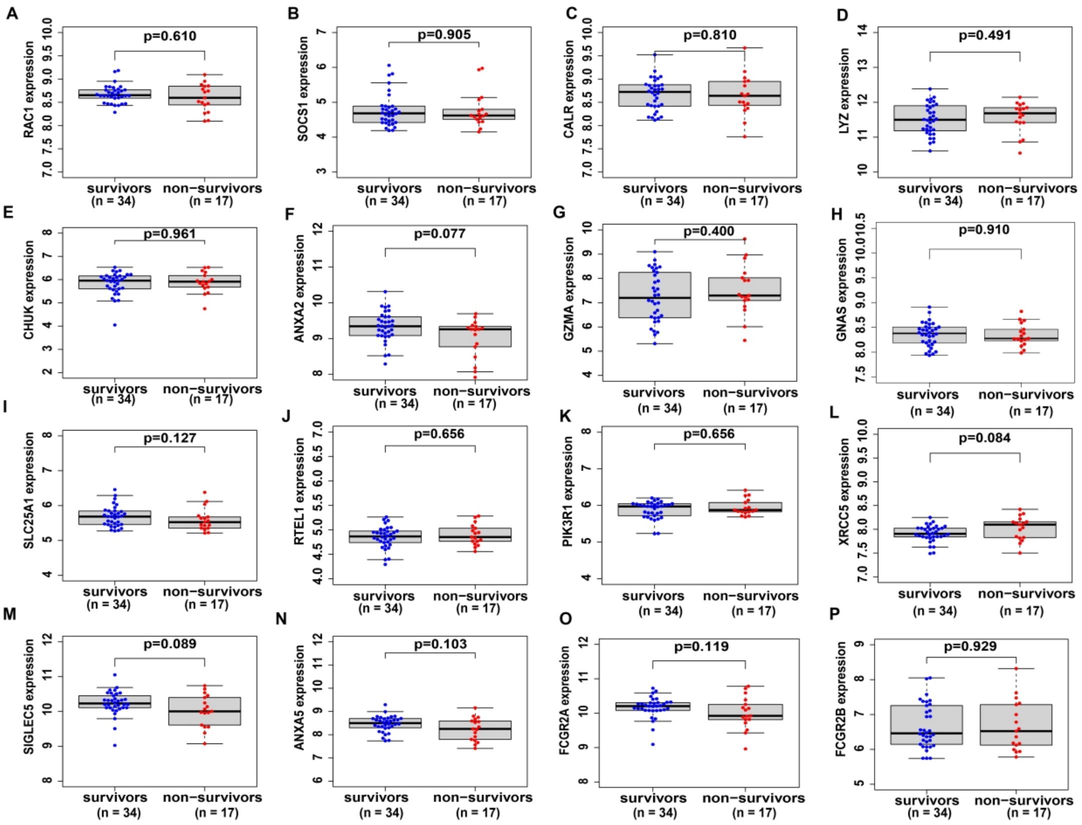

Figure S2 Verification of the expression of candidate genes(A-N), FCGR2A(O), and FCGR2B(P) in the first validation cohort (GSE95233). All genes showed no expression difference between septic survivors and non-survivors.

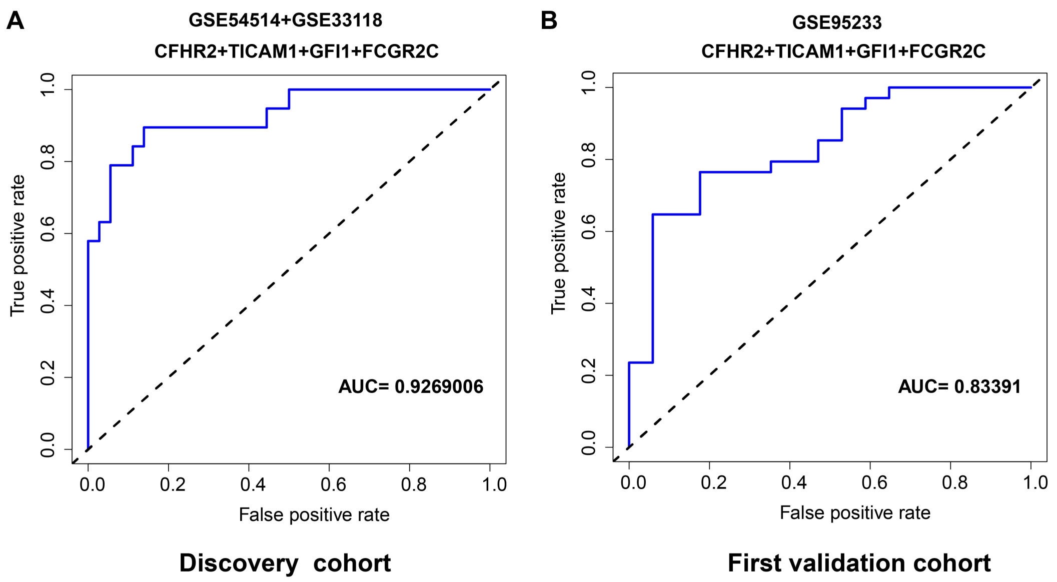

Figure S3 The prognostic evaluation ability of 4 genes (CFHR2, FCGR2C, GFI1, and TICAM1) in GEO datasets. (A) the discovery dataset (GSE54514+GSE33118). (B) the first validation dataset (GSE95233).

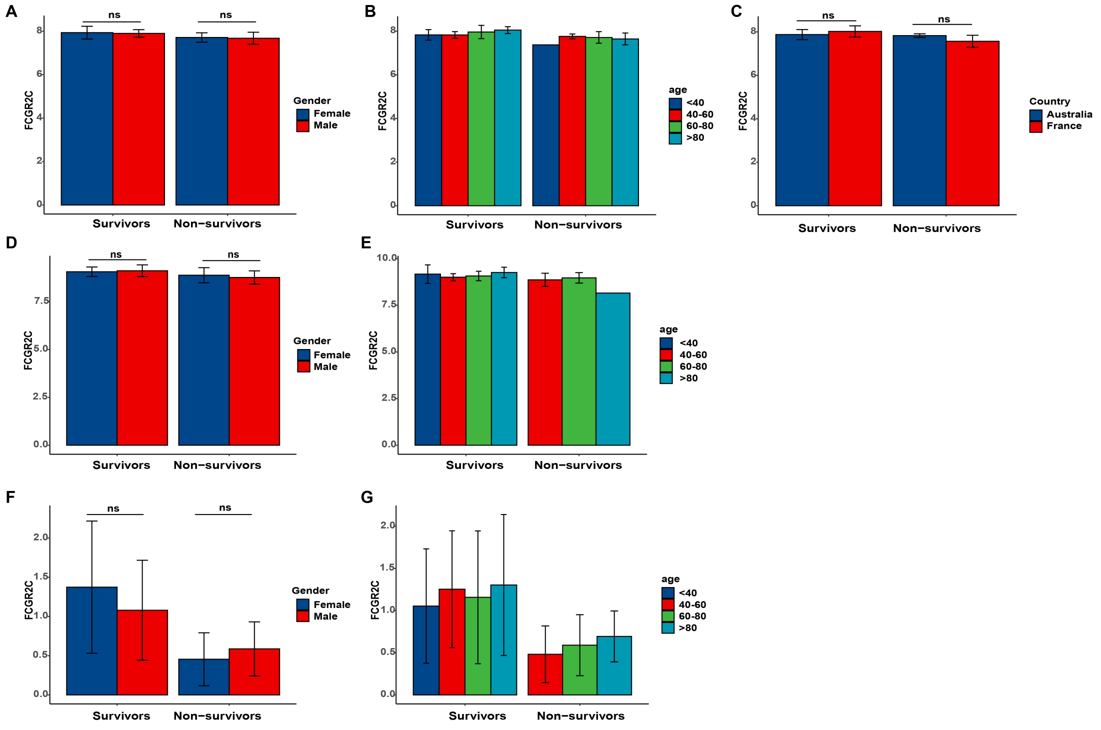

Figure S4 Expression levels of FCGR2C in different gender, age, and country groups. In the discovery cohort(GSE54514+GSE33118), the FCGR2C expression levels were in different gender(A), age(B), and country(C) groups. In the first validation dataset (GSE95233), the FCGR2C expression levels were in different gender(D), and age(E).

In the second validation cohort (our recruitment cohort), the FCGR2C expression levels were in different gender(F) and ages (G).

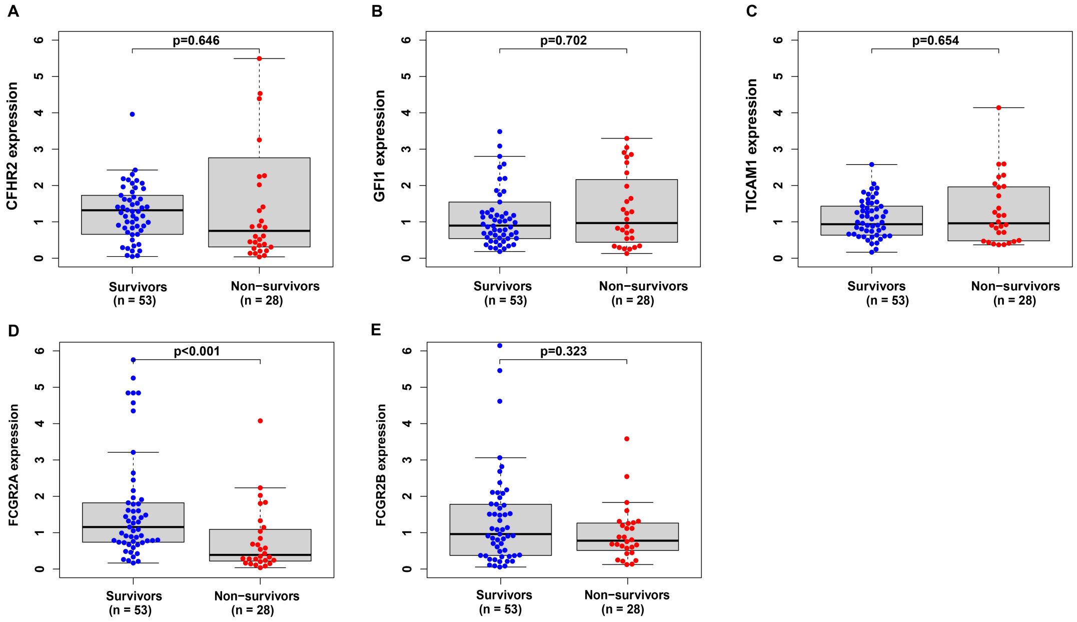

Figure S5 Verified the expression of CFHR2, GFI1, TICAM1, FCGR2A, and FCGR2B in the second validation cohort (our recruitment cohort).

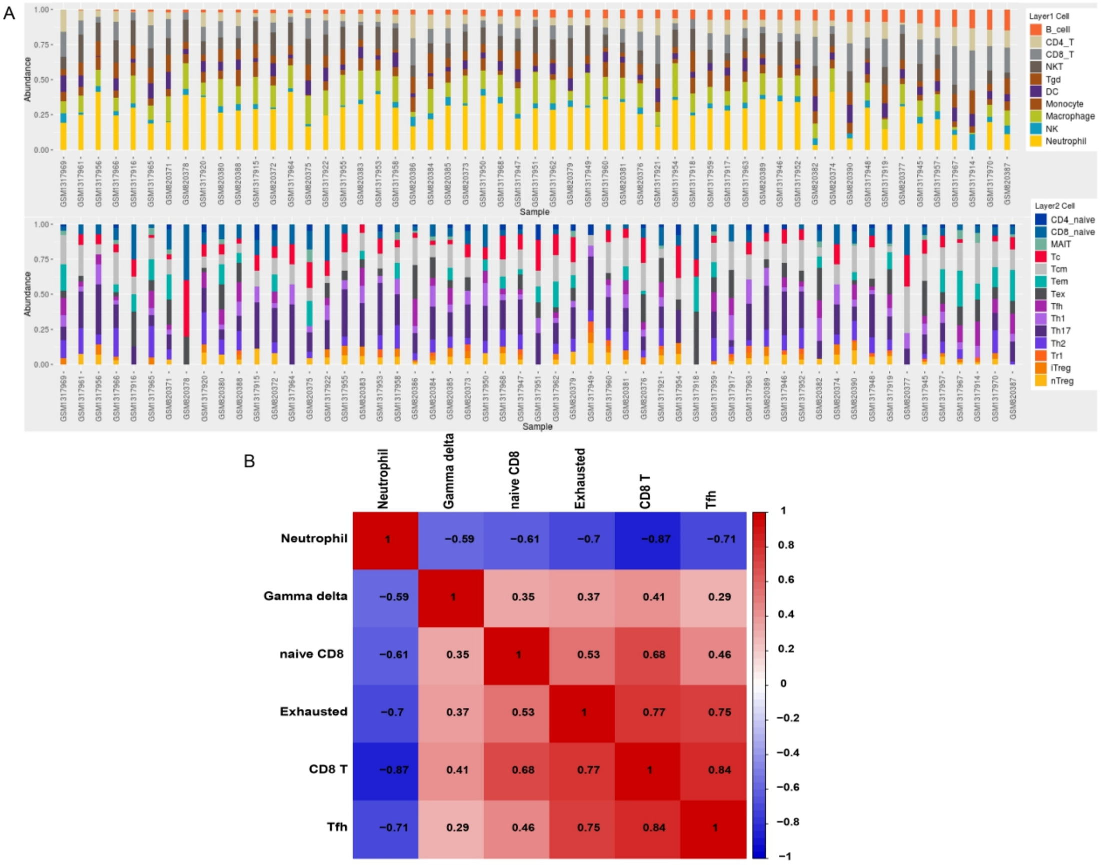

Figure S6 Relative abundances (A) and correlation heatmap (B) of immune cells by the ImmuCellAI analysis in the discovery cohort.

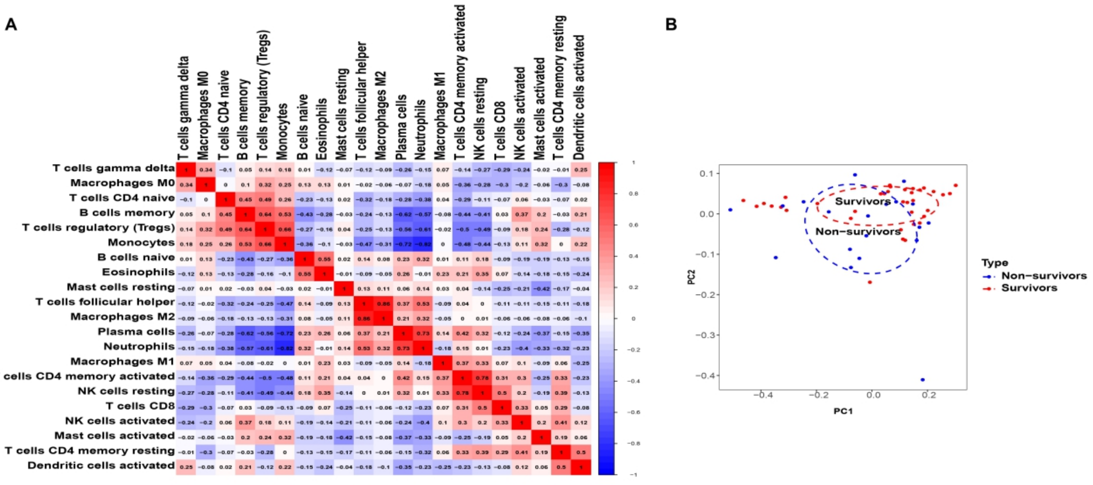

Figure S7 Correlation matrix of 22 immune cell compositions (A) and the PCA of 22 immune cells distinguish between septic survivors and non-survivors (B) by CIBERSORT analysis.

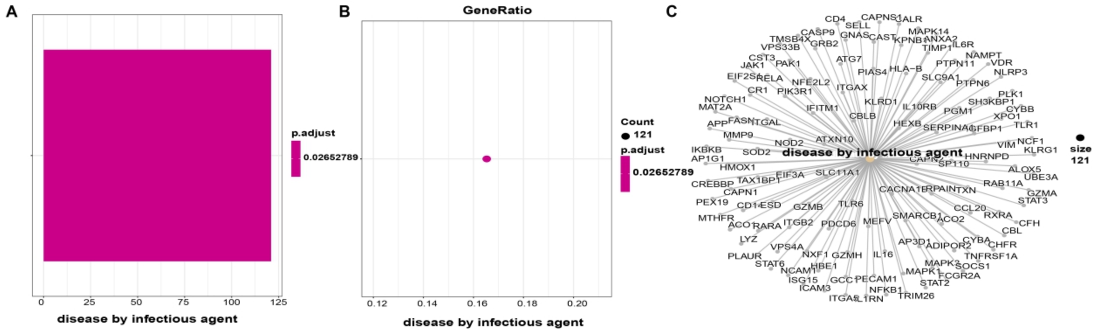

Figure S8 Disease Ontology (DO) enrichment analysis of DEGs in the discovery cohort. (A) Enrichment pathways with P-values less than 0.05 in DO enrichment analysis. (B) The number of genes enriched in disease by an infectious agent pathway. (C) A total of 121 genes were involved in the disease by an infectious agent pathway.
