# Supplementary material for: FCGR2C: An emerging immune gene for predicting sepsis outcome
Source: Front Immunol. 2022 Dec 2;13:1028785. doi: 10.3389/fimmu.2022.1028785 (PMC9757160; doi:10.3389/fimmu.2022.1028785)
Supplement: Supplementary file 2 [file Table_1.docx]

**Table S1 Basic information of the septic patients in public datasets.**

|  | **Datasets** | **Race** |  | | Survivors  (n=26) | Non-survivors  (n=9) | ***p*** |
| --- | --- | --- | --- | --- | --- | --- | --- |
| **The discovery cohort** | GSE54514 | White  (Australia) | Age, years(mean±SD) | | 56.69 (18.27) | 69.67 (11.01) | 0.062 |
|  |  |  | Gender (males/females) | | 16/10 | 5/4 | 0.77 |
|  |  |  | Infection  Site | Lung | 10 | 5 |  |
|  |  |  |  | Blood | 3 | 3 |  |
|  |  |  |  | Others | 9 |  |  |
|  |  |  |  | Unknown | 4 | 1 |  |
|  | GSE33118 | White  (France) |  | | Survivors  (n=10) | Non-survivors  (n=10) | ***p*** |
|  |  |  | Age, years(mean±SD) | | No specific | No specific |  |
|  |  |  | Gender (males/females) | | 6/4 | 6/4 | 1 |
|  |  |  | Infection Site | | Pneumonia | Pneumonia |  |
| **The first validation**  **cohort** | GSE95233 | White  (France) |  | | Survivors  (n=34) | Non-survivors  (n=17) | ***p*** |
|  |  |  | Age, years(mean±SD) | | 62.85 (16.21) | 60.88 (12.82) | 0.42 |
|  |  |  | Gender (males/females) | | 25/9 | 6/9 | 0.027 |
|  |  |  | Infection Site | | NA | NA |  |

In the GSE95233 cohort, the gender of 2 non-survivors patients was unclear.
