# Supplementary material for: FCGR2C: An emerging immune gene for predicting sepsis outcome
Source: Front Immunol. 2022 Dec 2;13:1028785. doi: 10.3389/fimmu.2022.1028785 (PMC9757160; doi:10.3389/fimmu.2022.1028785)
Supplement: Supplementary file 3 [file Table_2.docx]

**Table S2 The sequences of the gene-specific primers used in this study.**

| Species | Gene name | Base sequence |
| --- | --- | --- |
| Human | CFHR2 forward | TCATGTGTAGAACGGGGCTG |
| Human | CFHR2 reverse | AGGGGGCCCACATTTTTCTG |
| Human | FCGR2C forward | ACCATCACTGTCCAAGCTCC |
| Human | FCGR2C reverse | CTCAAATTGGGCAGCCTTCAC |
| Human | GFI1 forward | GACCCTTTGCCTGCGAGATGTG |
| Human | GFI1 reverse | GGACAGTGTGGATGACCTCTTGAAG |
| Human | TICAM1 forward | CACTTCCTAGCGCCTTCGAC |
| Human | TICAM1 reverse | GTGGGGTCTTCAGTTTGTGC |
| Human | β-actin forward | GAGAAAATCTGGCACCACACC |
| Human | β-actin reverse | GGATAGCACAGCCTGGATAGCAA |
| Human | FCGR2A forward | CTTCTGCAGACAGTCAAGCTG |
| Human | FCGR2A reverse | CCCCGCTGTCATTGTTGTTG |
| Human | FCGR2B forward | AGCCAATCCCACTAATCCTGA |
| Human | FCGR2B reverse | GGTGCATGAGAAGTGAATAGGTG |
