# Supplementary material for: FCGR2C: An emerging immune gene for predicting sepsis outcome
Source: Front Immunol. 2022 Dec 2;13:1028785. doi: 10.3389/fimmu.2022.1028785 (PMC9757160; doi:10.3389/fimmu.2022.1028785)
Supplement: Supplementary file 5 [file Table_4.docx]

|  | | Survivors  (n=53) | Non-survivors  (n=28) | *P* |
| --- | --- | --- | --- | --- |
| Age, years | | 62.94(15.17) | 66.07 (12.51) | 0.44 |
| Gender(males/females) | | 31/22 | 22/6 | 0.073 |
| Comorbidities, n | |  |  |  |
| Diabetes | | 17 | 10 | 0.79 |
| Hypertension | | 28 | 17 | 0.50 |
| Coronary heart disease | | 10 | 12 | 0.022 |
| Tumour | | 9 | 5 | 0.93 |
| Liver disease | | 12 | 10 | 0.21 |
| kidney disease | | 19 | 11 | 0.68 |
| Invasive ventilator | | 6 | 13 | <0.001 |
| Noninvasive ventilator | | 31 | 19 | 0.86 |
| Hemodialysis | | 14 | 18 | 0.001 |
| Antibiotics | | 53 | 28 |  |
| SARS-CoV-2(+/-) | | 53(-) | 28(-) |  |
| Infection site, n | Intestinal | 13 | 8 |  |
|  | Urinary | 11 | 6 |  |
|  | Respiratory | 13 | 6 |  |
|  | Skin and soft tissue | 11 | 4 |  |
|  | Others or unknown | 5 | 4 |  |
| SOFA score | | 6.28 (3.32) | 10.82 (4.15) | <0.001 |
| APACHEII score | | 15.98 (5.47) | 20.64 (7.31) | 0.0054 |
| GCS score | | 14.40 (1.88) | 10.93 (5.30) | <0.001 |
| IL-2 | | 0.96 (1.25) | 2.41 (3.50) | 0.021 |
| IL-6 | | 2747.4 (6171.8) | 5442.2 (6355.9) | <0.001 |
| IL-10 | | 127.10 (365.16) | 1189.51 (3063.4) | <0.001 |
| TNF-α | | 2.34 (5.61) | 9.91 (21.91) | <0.001 |
| Neutrophil | | 17.65 (10.04) | 12.54 (7.63) | 0.044 |
| CD3+ T cells | | 65.12 (14.22) | 53.52 (18.70) | 0.01 |
| CD8+ T cells | | 26.83 (13.2) | 21.46 (13.4) | 0.045 |
| Absolute value of T cells | | 537.16 (358.40) | 447.00 (566.51) | 0.047 |
| Absolute value of CD8+ T cells | | 228.22 (219.24) | 187.62 (342.15) | 0.027 |
| C3 | | 0.88 (0.26) | 0.72 (0.38) | 0.027 |

**Table S4 The clinical information of the recruited septic patients in this study.**

Continuous and classified variables were presented as mean ± standard deviation and number (percentages). (-): represents a negative SARS-CoV-2, (+): represents a positive SARS-CoV-2. Neutrophil: represents the absolute value of neutrophils. CD3+ T cells and CD8+ T cells: represent the percentage of CD3+ T cells and CD8+ T cells, respectively. Wilcoxon test was used to compare continuous variables, and the chi-square test was applied to compare categorical variables.

Sequential organ failure assessment (SOFA) score:

<https://www.mdcalc.com/calc/691/sequential-organ-failure-assessment-sofa-score>

Glasgow coma scale (GCS) score:

<https://www.mdcalc.com/calc/10140/gcs-pupils-score>

Acute physiology and chronic health evaluation II (APACHE II) score：

<https://www.mdcalc.com/calc/1868/apache-ii-score>
