# Supplementary material for: FCGR2C: An emerging immune gene for predicting sepsis outcome
Source: Front Immunol. 2022 Dec 2;13:1028785. doi: 10.3389/fimmu.2022.1028785 (PMC9757160; doi:10.3389/fimmu.2022.1028785)
Supplement: Supplementary file 7 [file Table_6.docx]

**Table S6 The sequencing results of qRT-PCR products**

FCGR2C-F-products(130 bp)：

CTTGGTGGGCTGTGGTCACTGGGATTGCTGTAGCGGCCATTGTTGCTGCTGTAGTGGCCTTGATCTACTGCAGGAAAAAGCGGATTTCAGCCAATTCCACTGATCCTGTGAAGGCTGCCCAATTTGAGA

FCGR2C-R-product(136 bp)：

TTGAGCTGAGTCCGCTTTTTCCTGCAGTAGATCAAGGCCACTACAGCAGCAACAATGGCCGCTACAGCAATCCCAGTGACCACAGCCACAATGATCCCCATCGGTGAAGAGCTGGGAGCTTGGACAGTGATGGTA

The sequence files after double-end splicing(171 bp):

TCTCAAATTGGGCAGCCTTCACAGGATCAGTGGAATTGGCTGAAATCCGCTTTTTCCTGCAGTAGATCAAGGCCACTACAGCAGCAACAATGGCCGCTACAGCAATCCCAGTGACCACAGCCACAATGATCCCCATCGGTGAAGAGCTGGGAGCTTGGACAGTGATGGTA
